# Supplementary material for: Novel Driver Strength Index highlights important cancer genes in TCGA PanCanAtlas patients
Source: PeerJ. 2022 Aug 11;10:e13860. doi: 10.7717/peerj.13860 (PMC9375969; doi:10.7717/peerj.13860)
Supplement: Supplemental Information 2 [file peerj-10-13860-s002.zip › COHORTS/genes plots/detailed/2021_8_16_14_9_distribution_events_detailed_23.pdf]

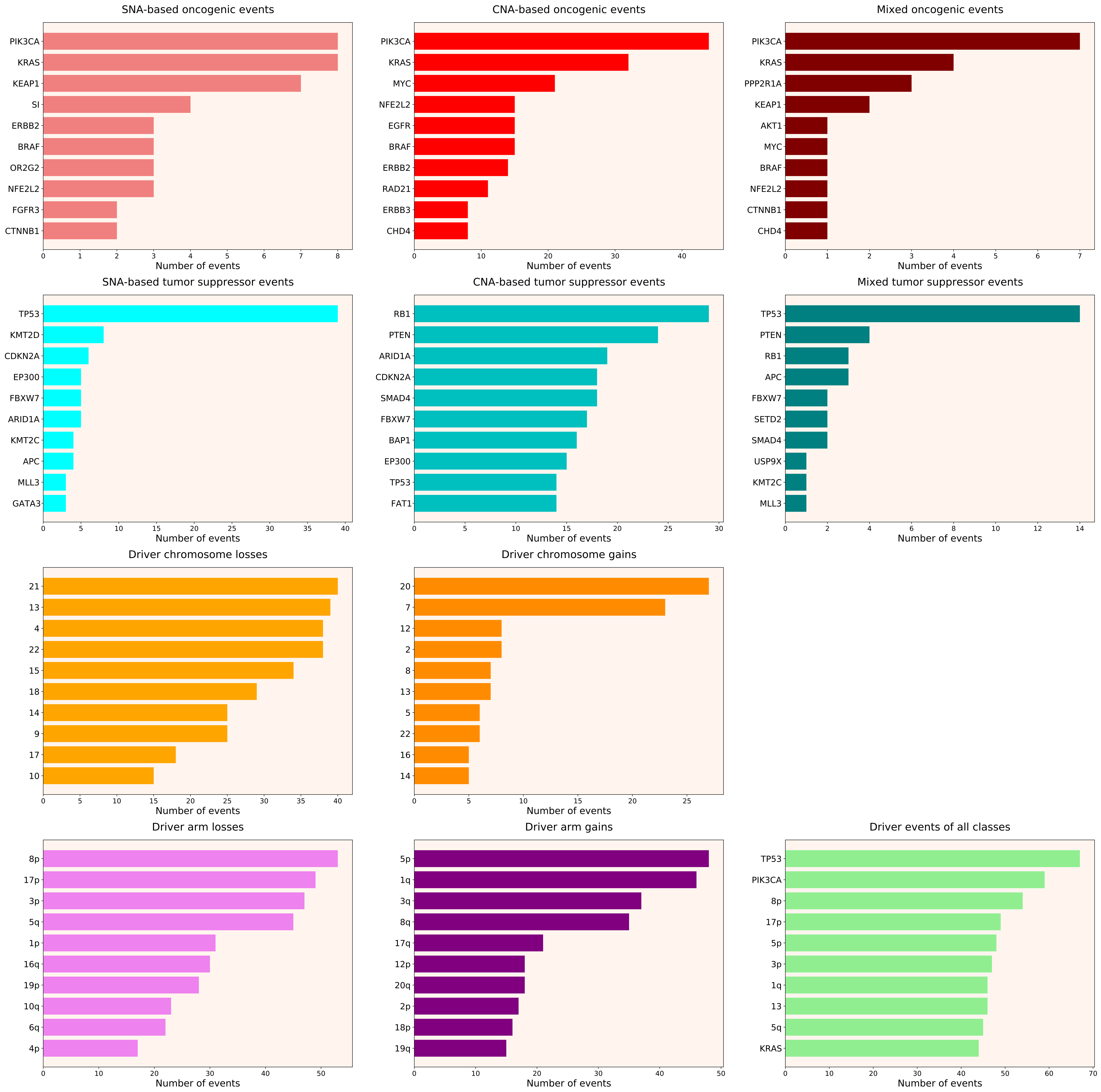
SNA-based oncogenic events
PIK3CA
KRAS
KEAP1
SI
ERBB2
BRAF
OR2G2
NFE2L2
FGFR3
CTNNB1
0 1 2 3 4 5 6 7 8
Number of events
CNA-based oncogenic events
PIK3CA
KRAS
MYC
NFE2L2
EGFR
BRAF
ERBB2
RAD21
ERBB3
CHD4
0 10 20 30 40
Number of events
Mixed oncogenic events
PIK3CA
KRAS
PPP2R1A
KEAP1
AKT1
MYC
BRAF
NFE2L2
CTNNB1
CHD4
0 1 2 3 4 5 6 7
Number of events
SNA-based tumor suppressor events
TP53
KMT2D
CDKN2A
EP300
FBXW7
ARID1A
KMT2C
APC
MLL3
GATA3
0 5 10 15 20 25 30 35 40
Number of events
CNA-based tumor suppressor events
RB1
PTEN
ARID1A
CDKN2A
SMAD4
FBXW7
BAP1
EP300
TP53
FAT1
0 5 10 15 20 25 30
Number of events
Mixed tumor suppressor events
TP53
PTEN
RB1
APC
FBXW7
SETD2
SMAD4
USP9X
KMT2C
MLL3
0 2 4 6 8 10 12 14
Number of events
Driver chromosome losses
21
13
4
22
15
18
14
9
17
10
0 5 10 15 20 25 30 35 40
Number of events
Driver chromosome gains
20
7
12
2
8
13
5
22
16
14
0 5 10 15 20 25
Number of events
Driver arm losses
8p
17p
3p
5q
1p
16q
19p
10q
6q
4p
0 10 20 30 40 50
Number of events
Driver arm gains
5p
1q
3q
8q
17q
12p
20q
2p
18p
19q
0 10 20 30 40 50
Number of events
Driver events of all classes
TP53
PIK3CA
8p
17p
5p
3p
1q
13
5q
KRAS
0 10 20 30 40 50 60 70
Number of events
